# Profiling the susceptibility of *Pseudomonas aeruginosa* strains from acute and chronic infections to cell-wall-targeting immune proteins

**DOI:** 10.1038/s41598-019-40440-w

**Published:** 2019-03-05

**Authors:** Gabriel Torrens, Isabel M. Barceló, Marcelo Pérez-Gallego, Maria Escobar-Salom, Sara Tur-Gracia, Marta Munar-Bestard, María del Mar González-Nicolau, Yoandy José Cabrera-Venegas, Estefany Nayarith Rigo-Rumbos, Gabriel Cabot, Carla López-Causapé, Estrella Rojo-Molinero, Antonio Oliver, Carlos Juan

**Affiliations:** Servicio de Microbiología and Unidad de Investigación, Hospital Universitari Son Espases-Institut de Investigació Sanitària de Balears (IdISBa), Palma, Spain

**Keywords:** Antimicrobial resistance, Pathogens

## Abstract

In the current scenario of high antibiotic resistance, the search for therapeutic options against *Pseudomonas aeruginosa* must be approached from different perspectives: cell-wall biology as source of bacterial weak points and our immune system as source of weapons. Our recent study suggests that once the permeability barrier has been overcome, the activity of our cell-wall-targeting immune proteins is notably enhanced, more in mutants with impaired peptidoglycan recycling. The present work aims at analyzing the activity of these proteins [lysozyme and Peptidoglycan-Recognition-Proteins (PGLYRPs)], alone or with a permeabilizer (subinhibitory colistin) in clinical strains, along with other features related to the cell-wall. We compared the most relevant and complementary scenarios: acute (bacteremia) and chronic infections [early/late isolates from lungs of cystic fibrosis (CF) patients]. Although a low activity of lysozyme/PGLYRPs *per se* (except punctual highly susceptible strains) was found, the colistin addition significantly increased their activity regardless of the strains’ colistin resistance levels. Our results show increased susceptibility in late CF isolates, suggesting that CF adaptation renders *P. aeruginosa* more vulnerable to proteins targeting the cell-wall. Thus, our work suggests that attacking some *P. aeruginosa* cell-wall biology-related elements to increase the activity of our innate weapons could be a promising therapeutic strategy.

## Introduction

*Pseudomonas aeruginosa* is one of the most important opportunistic pathogens and a very frequent cause of acute nosocomial and chronic infections in patients with underlying chronic respiratory diseases such as cystic fibrosis (CF)^1–3^. Its most striking feature is the outstanding capacity for antibiotic resistance development through the selection of chromosomal mutations and/or the horizontal acquisition of resistance determinants^4–6^. Increasing worldwide resistance levels compromise our therapeutic arsenal, making the finding of new targets essential, at least to make the infections less harmful for patients (antivirulence therapies)^7^. Amongst the lines searching for useful weak points in *P. aeruginosa* biology, some focus on the cell-wall and more specifically the peptidoglycan (PGN) and its metabolism, which is the target for β–lactams, the most used antipseudomonal drugs in clinical practice. In this regard, some works have proposed the pathways regulating the expression of the AmpC β–lactamase, intimately linked to the PGN metabolism, as targets to avoid/revert β–lactam resistance in *P*. aeruginosa^8–12^. Additionally, many different elements have been proposed in the gram-negatives’ PGN biology as therapeutic targets, mainly in the field of anti-virulence^13^. In this context, we have recently shown that *P. aeruginosa* PGN recycling exerts a key protective role against the immune proteins targeting this structure [lysozyme and PGN Recognition Proteins (PGLYRPs)^14^]. A low activity of lysozyme or PGLYRPs was shown against the reference strain PAO1 but an important increase was found when a well-known permeabilizing agent (colistin)^15^ was added at subinhibitory concentrations. Moreover, this effect was dramatically enhanced in mutants with impaired PGN recycling^14^.

Therefore, this work was aimed at analyzing the activity of these proteins *per se* or combined with subinhibitory colistin in clinical strains for the first time. We analyzed the two most relevant and complementary scenarios, acute infections (bloodstream isolates) and chronic infections (early/late isolates from lungs of CF patients). It is well known that *P. aeruginosa* accumulates multiple adaptive mutations, such as those leading to virulence attenuation or antimicrobial resistance, that promote persistence in the CF lung niche, where bacteria are notably protected against the diffusion of certain immune elements mainly thanks to the mucus and biofilm lifestyle^16–18^. This scenario is completely different to that the bloodstream demands for infection development, in which acute virulence is determinant^19^. Thus, we aimed to search for the correlations between the characterized phenotypes and additional cell wall-linked features to explore the potential implications of these circumstances and the obtained data. Among these features, the susceptibility against serum and β-Defensin 1 and accessory traits weaklier related with the cell-wall, such as the inflammatory and cytotoxic capacities, were analyzed. Altogether, our results show that attacking some of the *P. aeruginosa* cell-wall biology-related elements to increase the activity of our innate humoral weapons could be a very promising therapeutic strategy. Moreover, our results draw a complex scenario revealing the participation of many actors ultimately defining the phenotypes of the clinical strains in terms of their susceptibility to the studied innate weapons, which opens a new horizon in the search for targets to habilitate these elements as future antipseudomonal resources.

## Results

### Lysozyme and PGLYRPs show a modest *in vitro* activity *per se* on clinical strains, with punctual exceptions

**Figure 1:**
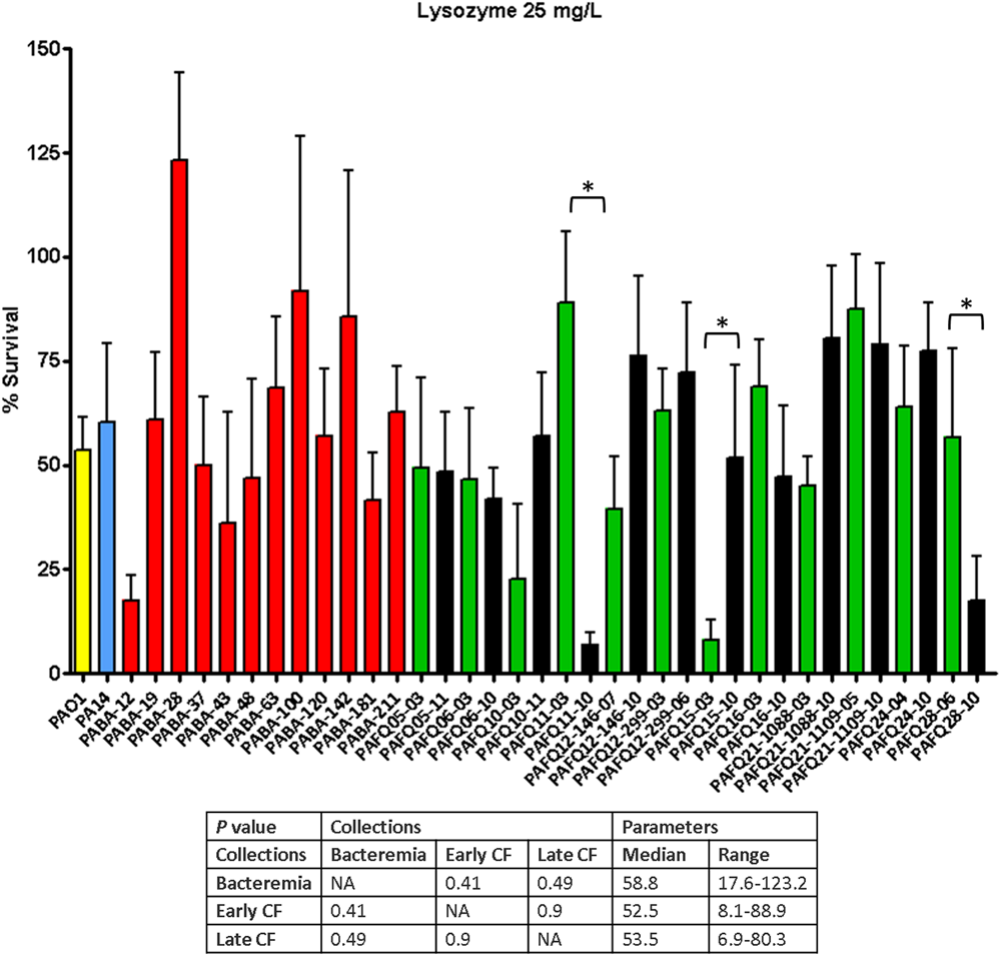
Survival rates of the *P. aeruginosa* clinical strains after treatment with lysozyme 25 mg/L. Incubation with lysozyme was performed as explained in materials and methods (1 × 10^6^ CFUs of each strain, 37 °C, 180 r.p.m. agitation, 1 h), and the survival percentage was calculated with regards to the initial inoculum. Each column represents the mean value of at least three independent assays for each specific strain, whereas the error bar represents the standard deviation (SD). In the CF pairs of isogenic isolates, the green columns correspond to early and the black to late isolates, respectively. All the bacteremia strains are displayed with red columns. The asterisks over the bars indicate a statistically significant difference between the early/late isolates in the specific pair(s) of CF strains, *P* < 0.05 in the One-way ANOVA with post hoc Tukey’s multiple comparison test. The differences among bacteria treated with lysozyme and controls (bacteria incubated in the assay buffer without protein) were statistically significant in all the cases, with a slight growth in the control tubes (bacterial survival > 100%, *P* < 0.05, data not shown). The box below displays the statistical parameters obtained when analyzing the outcomes of the strains grouping them in the three collections (bacteremia, early CF isolates and late CF isolates). The performed *t* tests were two-tailed in all the cases; only when comparing the early with late CF isolates, a paired test was applied. *A *P* value < 0.05 was considered statistically significant regarding the differences among the mean values of collections. NA: Not applicable.

As can be observed in Fig. 1 and the Supplementary DataSet S1, the overall activity of lysozyme in the three collections (bacteremia, early CF and late CF isolates) was modest, with mean survival percentages circa 50–60%, very similar to those of the reference strains PAO1 and PA14 (54% and 60%). Even though the One-way ANOVA test indicated that the susceptibility behaviors were heterogeneous, when comparing the survival percentages among the collections, the differences were never statistically significant (*P* > 0.05). Nevertheless, some strains stood out due to their high susceptibility, with survival rates well below the mean values, e.g. PAFQ15-3 or PAFQ11-10, both below 10%. When comparing the pairs of CF early/late isolates, the differences were statistically significant (One-way ANOVA with *post hoc* Tukey’s test) in only three of them, but with no clear trend regarding susceptibility overtime.

**Figure 2:**
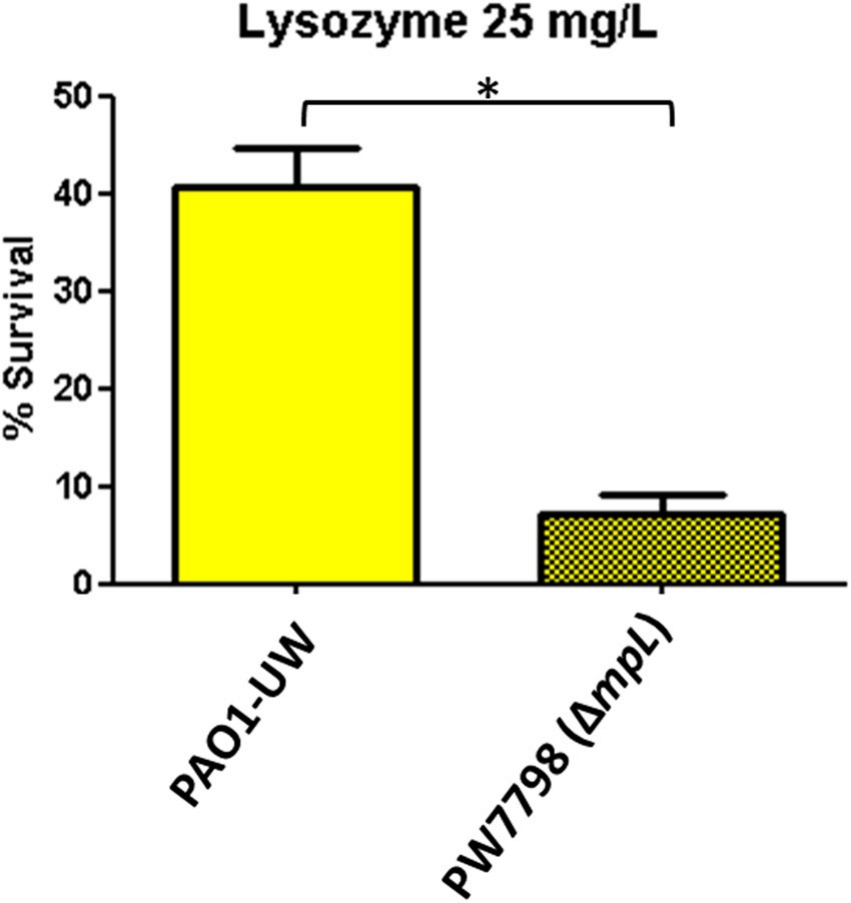
Bacterial survival after treatment with lysozyme 25 mg/L, in the *mpL* knockout mutant (PW7798) from the UW Library^22^, and its originary wildtype strain (PAO1-UW). Incubations were performed as explained in materials and methods (1 × 10^6^ CFUs of each strain, 37 °C, 180 r.p.m. agitation, 1 h), and the survival percentage was calculated with regards to the initial inoculum. Each column represents the mean value of at least three independent assays for each strain, whereas the error bar represents the standard deviation (SD). *Statistically significant differences between the two strains: *P* < 0.05 in the Student’s *t* test. The differences among bacteria treated with lysozyme and controls (bacteria incubated in the assay buffer without protein) were statistically significant in all the cases, with a slight growth in the control tubes (bacterial survival > 100%, *P* < 0.05, data not shown).

Although investigation of the genetic basis for the eventual attention-calling phenotypes was not a major objective of this study, some clues can be given for the increased susceptibility of isolate PAFQ11-10 to lysozyme. In this sense, a comparison of the published genomes of the pair PAFQ11^20^ revealed the mutational inactivation (premature stop codon) of the gene PA4010 (*mpl*) in PAFQ11-10, among other mutations. Since this gene is related with PGN recycling^21,22^, it was considered as potentially responsible for lysozyme susceptibility. Therefore, we tested the susceptibility of the *mpl* KO mutant from the University of Washington (UW) Library^23^. Our results, displayed in Fig. 2, suggest that *mpl* disruption seems clearly involved, since the bacterial survival decreased to less than a fifth in the *mpl* mutant, compared to wildtype (*P* < 0.0001).

Finally, the overall activity of PGLYRPs was even lower than that of lysozyme, with mean survival rates of circa 80%, very uniform among all the strains (with no statistically significant differences among collections, Supplementary Fig. S1). Therefore, we found no phenotypes of high susceptibility to PGLYRPs, or trends when comparing CF initial/late isolates.

### Subinhibitory colistin significantly enhances the activity of lysozyme/PGLYRPs, with greater effect on cystic fibrosis than on the bacteremia isolates regardless of their minimum inhibitory concentrations

**Figure 3:**
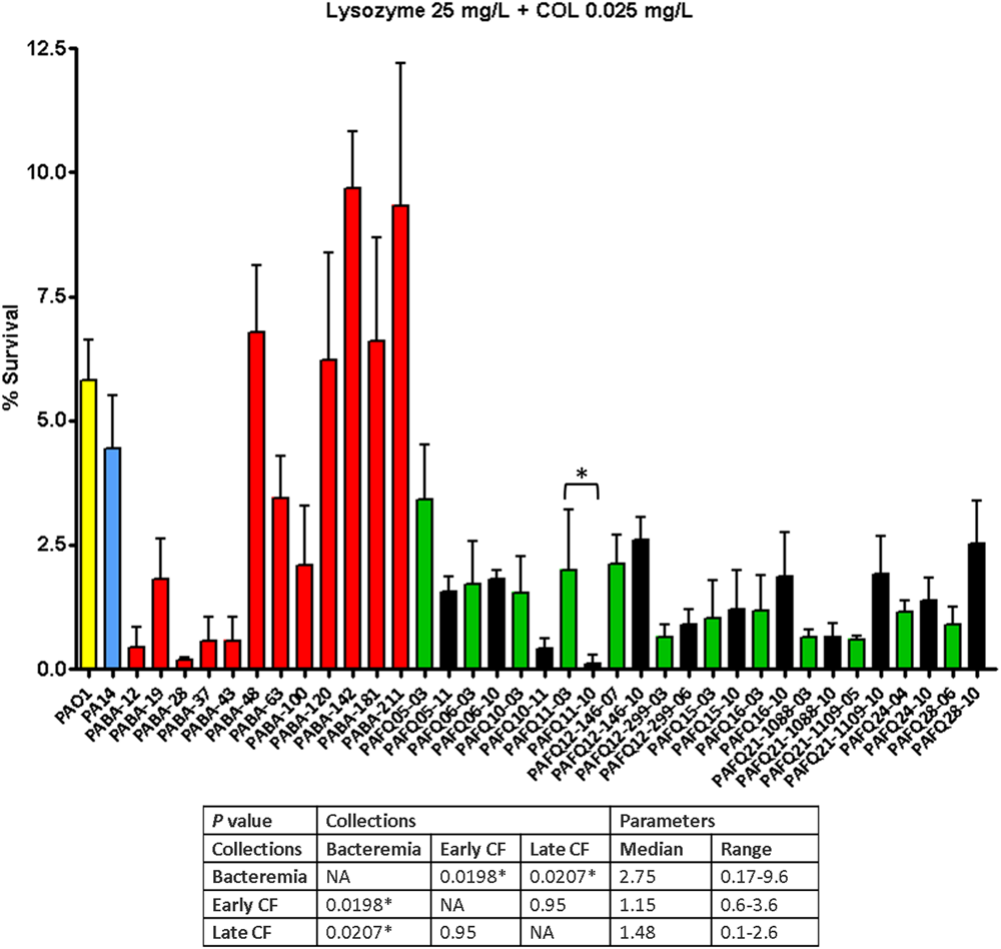
Survival rates of the *P. aeruginosa* clinical strains after treatment with lysozyme + colistin (25 mg/L + 0.025 mg/L). Incubation was performed as explained in materials and methods (1 × 10^6^ CFUs of each strain, 37 °C, 180 r.p.m. agitation, 1 h), and the survival percentage was calculated with regards to the initial inoculum. Each column represents the mean value of at least three independent assays for each specific strain, whereas the error bar represents the standard deviation (SD). In the CF pairs of isogenic isolates, the green columns correspond to early and the black to late isolates, respectively. All the bacteremia strains are displayed with red columns. The asterisks over the bars indicate a statistically significant difference between the early/late isolates in the specific pair(s) of CF strains, *P* < 0.05 in the One-way ANOVA with post hoc Tukey’s multiple comparison test. The differences among bacteria treated with lysozyme + colistin and controls (bacteria incubated in the assay buffer without protein) were statistically significant in all the cases, with a slight growth in the control tubes (bacterial survival > 100%, *P* < 0.05, data not shown). The box below displays the statistical parameters obtained when analyzing the outcomes of the strains grouping them in the three collections (bacteremia, early CF isolates and late CF isolates). The performed *t* tests were two-tailed in all the cases; only when comparing the early with late CF isolates, a paired test was applied. *A *P* value < 0.05 was considered statistically significant regarding the differences among the mean values of collections. NA: Not applicable.

**Figure 4:**
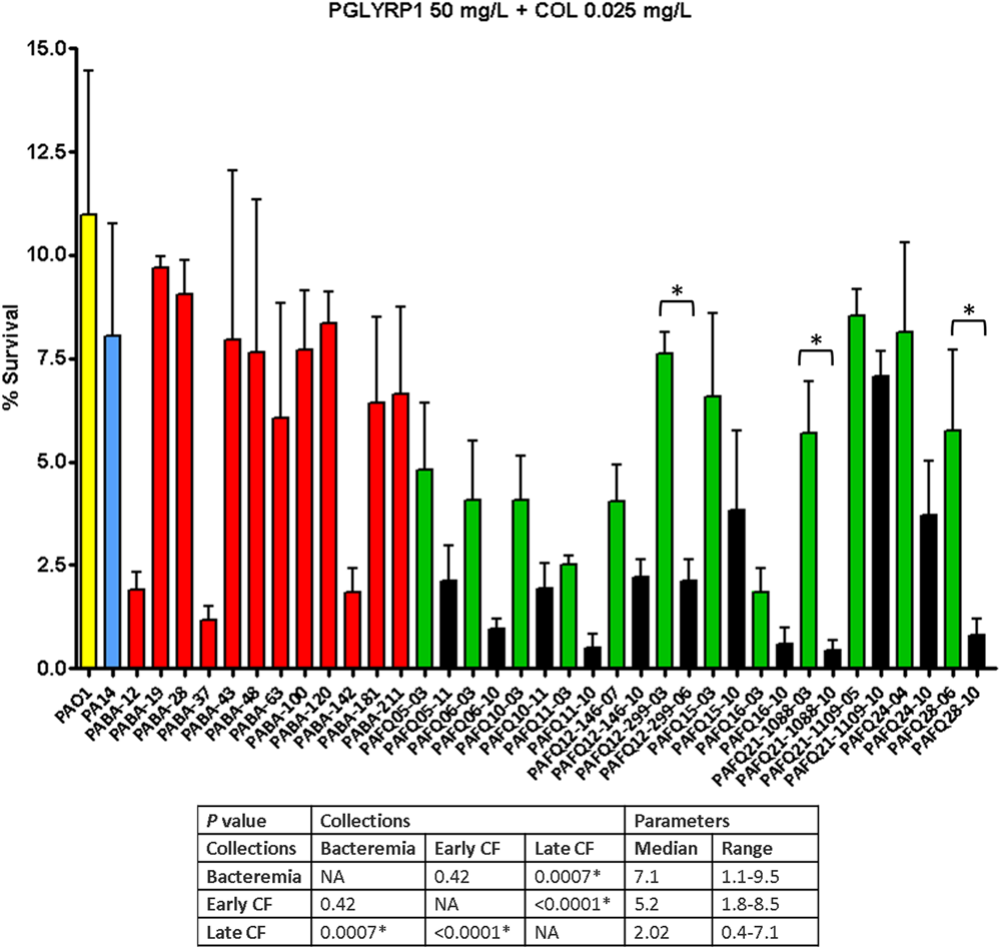
Survival rates of the *P. aeruginosa* clinical strains studied after treatment with PGLYRP1 + colistin (50 mg/L + 0.025 mg/L). Incubation was performed as explained in materials and methods (1 × 10^5^ CFUs of each strain, 2 h at 180 r.p.m., 37 °C), and the survival percentage was calculated with regards to the initial inoculum. Each column represents the mean value of at least three independent assays for each specific strain, whereas the error bar represents the standard deviation (SD). In the CF pairs of isogenic isolates, the green columns correspond to early and the black to late isolates, respectively. All the bacteremia strains are displayed with red columns. The asterisks over the bars indicate a statistically significant difference between the early/late isolates in the specific pair(s) of CF strains, *P* < 0.05 in the One-way ANOVA with post hoc Tukey’s multiple comparison test. The differences among bacteria treated with PGLYRP1 + colistin and controls (bacteria incubated in the assay buffer without protein) were statistically significant in all the cases, with a slight growth in the control tubes (bacterial survival > 100%, *P* < 0.05, data not shown). The box below displays the statistical parameters obtained when analyzing the outcomes of the strains grouping them in the three collections (bacteremia, early CF isolates and late CF isolates). The performed *t* tests were two-tailed in all the cases; only when comparing the early with late CF isolates, a paired test was applied. *A *P* value < 0.05 was considered statistically significant regarding the differences among the mean values of collections. NA: Not applicable.

**Figure 5:**
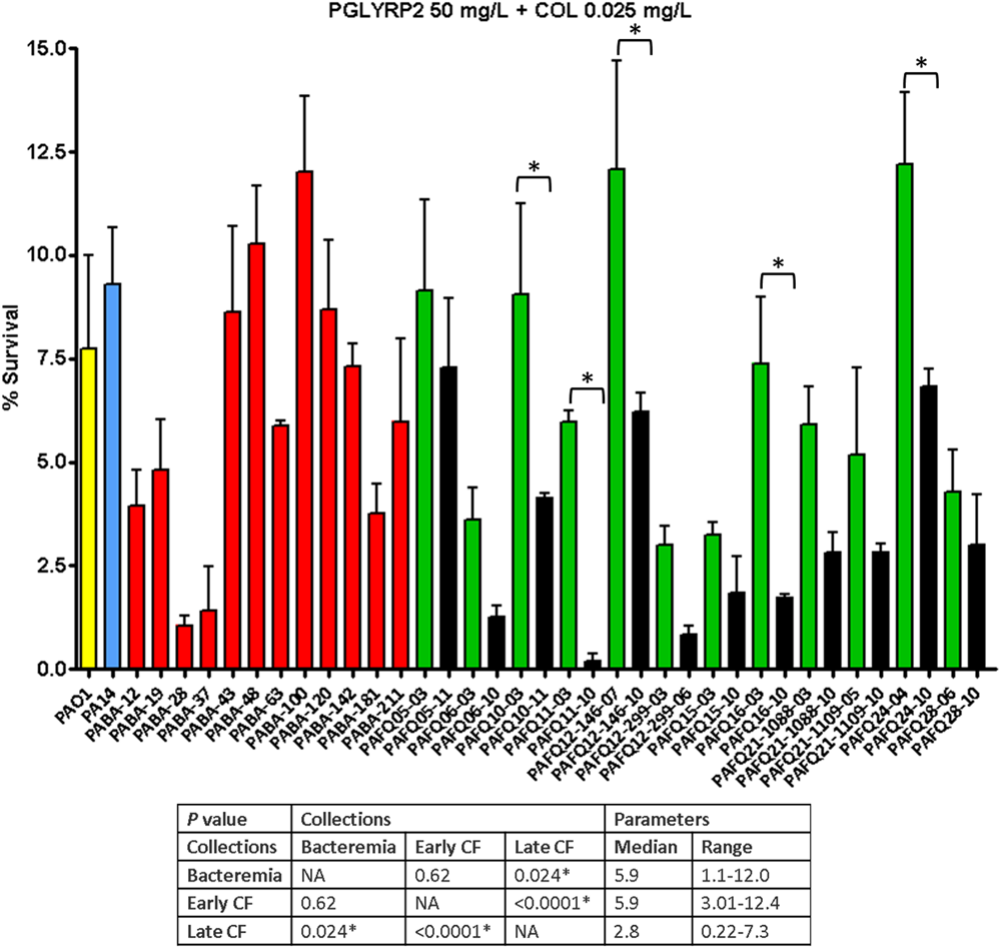
Survival rates of the *P. aeruginosa* clinical strains studied after treatment with PGLYRP2 + colistin (50 mg/L + 0.025 mg/L). Incubation was performed as explained in materials and methods (1 × 10^5^ CFUs of each strain, 2 h at 180 r.p.m., 37 °C), and the survival percentage was calculated with regards to the initial inoculum. Each column represents the mean value of at least three independent assays for each specific strain, whereas the error bar represents the standard deviation (SD). In the CF pairs of isogenic isolates, the green columns correspond to early and the black to late isolates, respectively. All the bacteremia strains are displayed with red columns. The asterisks over the bars indicate a statistically significant difference between the early/late isolates in the specific pair(s) of CF strains, *P* < 0.05 in the One-way ANOVA with post hoc Tukey’s multiple comparison test. The differences among bacteria treated with PGLYRP2 + colistin and controls (bacteria incubated in the assay buffer without protein) were statistically significant in all the cases, with a slight growth in the control tubes (bacterial survival > 100%, *P* < 0.05, data not shown). The box below displays the statistical parameters obtained when analyzing the outcomes of the strains grouping them in the three collections (bacteremia, early CF isolates and late CF isolates). The performed *t* tests were two-tailed in all the cases; only when comparing the early with late CF isolates, a paired test was applied. *A *P* value < 0.05 was considered statistically significant regarding the differences among the mean values of collections. NA: Not applicable.

**Figure 6:**
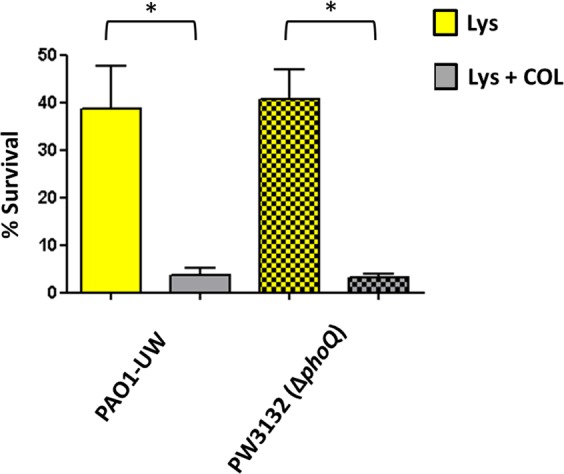
Bacterial survival after treatment with lysozyme (25 mg/L) or lysozyme plus colistin (25 mg/L + 0.025 mg/L), in the *phoQ* knockout mutant (PW3132) from the UW Library^22^, and its originary wildtype strain (PAO1-UW). Incubations were performed as explained in materials and methods, and the survival percentage was calculated with regards to the initial inoculum. Each column represents the mean value of at least three independent assays for each strain, whereas the error bar represents the standard deviation (SD). *Statistically significant differences between the two treatments: *P* < 0.05 in the Student’s *t* test. The differences among bacteria treated with lysozyme and controls (bacteria incubated in the assay buffer without protein) were statistically significant in all the cases, with a slight growth in the control tubes (bacterial survival > 100%, *P* < 0.05, data not shown).

As can be observed in Figs 3–5 and the Supplementary DataSet S1, the overall activity increase of the combined treatments (lysozyme/PGLYRPs + colistin) was very important in the majority of the strains regardless of the collection. The colistin concentration used (0.025 mg/L) was sub-inhibitory, since it was fairly well below the minimum inhibitory concentrations (MICs) of all the strains (the lowest MIC was 0.12 mg/L, Supplementary DataSet S1). As can be observed in this DataSet, where we display the fold-reduction in bacterial survival of each combined treatment with regards to incubation with the corresponding protein alone, virtually all the strains showed values at least at the reference strains levels (circa 10-fold). Nevertheless, the activity empowerment was quite variable: in the case of lysozyme + colistin, the ranges of fold-reduction in viability were 5.8–821.3 for bacteremia, and 6.9–145.5 for CF isolates. In the case of PGLYRP1 + colistin, the ranges were 8.8–78.1 (bacteremia) and 10.1–216.9 (CF), and regarding PGLYRP2 + colistin, the ranges were 6.5–63.7 (bacteremia) and 7.5–301.4 (CF). Thus, the activity increase was outstandingly high for some of the strains/treatments, such as PABA-28 or PAFQ21-1109-05 (lysozyme), PAFQ21-1088-10 (PGLYRP1) or PAFQ11-10 (PGLYRP2) (Supplementary DataSet S1). The reason some strains showed outstanding viability decreases for some of the combined treatments still remains to be ascertained. However, an obvious explanation could be the colistin susceptibility of each strain and therefore, in this case, some correlation between the MICs and the activity empowerment with colistin should exist. But our results suggest that the activity empowerment was not linked to the MICs: a correlation between the bactericidal and the permeabilizing activities of colistin was not seen. In fact, some strains showing clinical colistin resistance levels (MIC = 8 mg/L) such as PABA-48 or PAFQ12-146-07 showed no decreased empowerment of the combined treatment. Likewise, some of the strains with the lowest MICs, such as PAFQ15-10 or PABA-120 (0.12 mg/L), were not among those with the higher decreases in survival after adding colistin. To confirm these clues, and to determine whether resistance to colistin could impair the combined treatments, we incorporated the *phoQ* KO mutant from the UW Transposon Library^23^. *phoQ* inactivation has been shown to cause colistin resistance in *P. aeruginosa*^24–26^, and be inactivated in the strain PAFQ12-146-07 (MIC = 8 mg/L)^27^. Thus, we used the lysozyme and lysozyme + colistin treatments as indicators, comparing the *phoQ* mutant behavior with that of wildtype. As can be observed in Fig. 6, the decrease in survival when comparing lysozyme with the combined treatment was circa 10-fold both in PAO1^14^ and the mutant. Therefore, colistin resistance mediated by *phoQ* inactivation does not seem to interfere with the lysozyme activity empowerment provided by the subinhibitory colistin.

Regarding the descriptive statistics on the combined treatments, our results draw a clear trend separating the acute strains from those of CF. As observed in Fig. 3 and the Supplementary DataSet S1, a significantly higher susceptibility for lysozyme + colistin was found for CF strains (no differences between early/late isolates) compared with those from bacteremia: a circa three-fold decrease in survival. A similar trend was found for PGLYRPs + colistin treatments (Supplementary DataSet S1 and Figs 4 and 5), with a particular feature: the CF late isolates were significantly more susceptible than the early ones (survival decreases between two/three-fold). Another interesting fact regarding the different combined treatments is that the level of activity increase was often not proportional amongst themselves. For instance, the strain PABA-28, with an outstanding decrease in survival for lysozyme + colistin (circa 800-fold), showed a survival against PGLYRP1 + colistin at the level of reference strains. Conversely, there were some strains showing especially high susceptible phenotypes against the three combined treatments, as we recently described for mutants with impaired PGN recycling^14^: the strains PABA-12 and PABA-37, as well as the CF isolate PAFQ11-10, with survival percentages ≤ 0.3% for the three combinations in this latter strain (Supplementary DataSet S1). To gain insight into the basis for these different degrees of susceptibility to lysozyme/PGLYRPs (*per se* or with colistin), we studied the capacity of our strains’ cell-walls to counteract the osmotic pressure. It has been described that defects in the cell-wall metabolism render a weakened capacity to resist the hypo-osmotic shock^28,29^. But as can be observed in Supplementary Fig. S2, no direct cause-effect relatedness between osmotic shock susceptibility and the previously highlighted phenotypes could be found. This suggests that a simple structural weakening of the cell-wall would not be the most likely explanation for the enhanced susceptibilities to the studied treatments.

### Cystic fibrosis isolates are more susceptible against serum but not against β-Defensin 1 in comparison with bacteremia strains

To ascertain if the results displayed above could be related with additional cell-wall features, we characterized our collections in terms of susceptibility to other immune weapons targeting this structure: complement system (non-immune human serum is typically used for the study of the complement-mediated killing^30,31^) and β–Defensin 1.

**Figure 7:**
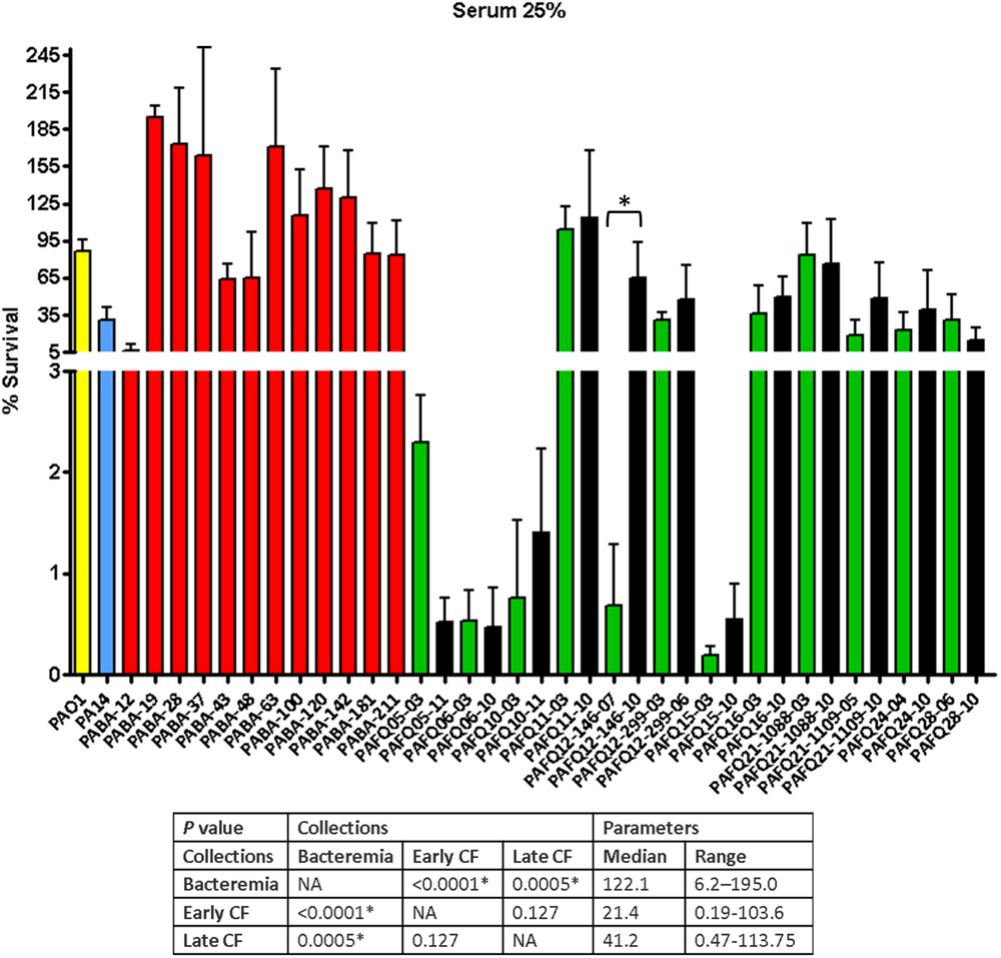
Survival rates of the *P. aeruginosa* clinical strains studied after treatment with 25% human fresh non-immune serum. Incubation was performed as explained in materials and methods (approximately 2 × 10^6^ CFUs of each strain, 37 °C, 30 minutes), and the survival percentage was calculated with regards to samples in which the serum was inactive (heat-inactivated). Each column represents the mean value of at least three independent assays for each specific strain, whereas the error bar represents the standard deviation (SD). In the CF pairs of isogenic isolates, the green columns correspond to early and the black to late isolates, respectively. All the bacteremia strains are displayed with red columns. The asterisks over the bars indicate a statistically significant difference between the early/late isolates in the specific pair(s) of CF strains, *P* < 0.05 in the One-way ANOVA with post hoc Tukey’s multiple comparison test. The differences among bacteria treated with 25% serum and controls (bacteria incubated in the assay buffer with 25% heat-inactivated serum) were statistically significant in all the cases, with a slight growth in the control tubes (bacterial survival > 100%, *P* < 0.05, data not shown). The box below displays the statistical parameters obtained when analyzing the outcomes of the strains grouping them in the three collections (bacteremia, early CF isolates and late CF isolates). The performed *t* tests were two-tailed in all the cases; only when comparing the early with late CF isolates, a paired test was applied. *A *P* value < 0.05 was considered statistically significant regarding the differences among the mean values of collections. NA: Not applicable.

The CF strains were bodily more serum-susceptible than those from bacteremia (Fig. 7 and Supplementary DataSet S1), although no trend when comparing the early/late isolates was evidenced. These results were expected since it has been reported that CF niche adaptation often renders strains with shorter or even absent LPS O-antigen chains, known to be usually serum-sensitive^30–32^. Nevertheless, the lack of O-antigen (driving to a non-typeable strain) did not necessarily involve serum susceptibility: i.e., the isolates from pairs PAFQ24 and PAFQ28 (all of them non-typeable) were much more serum-resistant than those from the pair PAFQ05 (serotype O1), showing high susceptibility (<2.5% of survival, Supplementary DataSet S1).

Regarding the activity of β-Defensin 1, published data suggest that the different variants of this cationic peptide are highly effective bactericidal weapons^33–36^ that can also be observed in our results: the overall activity was very high with no significant differences among collections (Supplementary Fig. S3 and Supplementary DataSet S1). In fact, the β-Defensin 1 treatment caused a higher reduction in bacterial viability among the treatments studied in this work. Moreover, what can be also deduced from our results is that a correlation between susceptibility levels to serum and defensin did not exist: some strains showing serum resistance were very susceptible to defensin (such as the majority of bacteremia strains) or vice-versa. For these reasons, an interesting issue could be to find out the basis for the increased survival rate of some of the strains (PABA-142, PABA-211, PAFQ10-11, PAFQ11-10 or the pair PAFQ24) against defensin, which could reveal clues to understand the exact mechanism(s) of action of this family of immune weapons, which is still a controversial issue^37,38^.

### Lack of association between profiles of susceptibility against immune proteins targeting the cell-wall and cytotoxic/inflammatory capacities

Finally, we attempted to identify correlations between the phenotypes described in the previous sections and additional markers more weakly related to the cell envelopes, but highly relevant for pathogenesis, such as the cytotoxicity or inflammatory capacity. Our results showed that overall the bacteremia strains were more cytotoxic than the CF ones. This analysis was statistically significant compared with late CF isolates but not with early ones (Supplementary Fig. S4, Supplementary DataSet S1). Indeed, it is widely accepted that the loss of virulence is a hallmark of CF strains that tends to be selected during adaptation to CF niche^39–44^. With regards to the elicited inflammatory response (Supplementary Fig. S5, Supplementary DataSet S1), the CF strains were overall more inflammatory than those from bacteremia (although with no statistical significance), which is in agreement with works claiming that, although adaptation to CF renders less virulent strains, their pro-inflammatory capacity remains very high, likely contributing to pathogenesis^2,45–47^. In any case, in the light of our findings and these previous works, what could be deduced is that the inflammatory response elicited by CF or acute strains is highly variable and that a general trend cannot be easily drawn.

**Figure 8:**
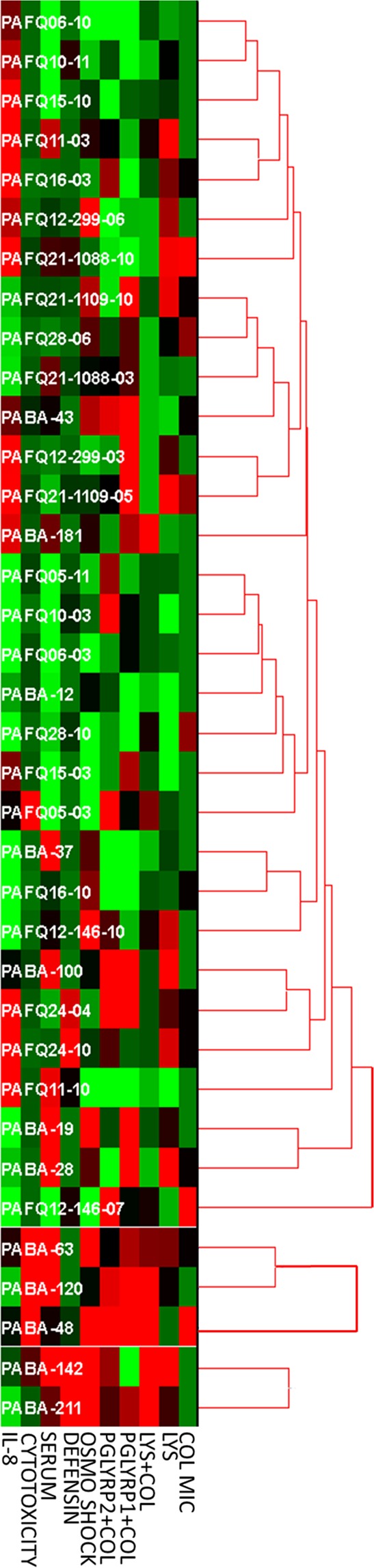
Hierarchical clustering of the strains according to the 10 measured variables showing statistically significant differences among strains (thus, the PGLYRPs treatments alone were not included). The values were normalized by Z-score, and the unsupervised hierarchical clustering (UPGMA, Euclidean distance, Minimum Similarity value = 0.5) was performed using the software HCE 3.5, available at http://www.cs.umd.edu/hcil/hce/ (University of Maryland). The lighter green and red are represented the squares, the lower and higher than the Z-score are the values, respectively. Therefore, the closer to black, the closer to 0 is the Z-score.

Finally, a search for correlations by hierarchical clustering among strains/phenotypes was performed, with the results being displayed in Fig. 8. As can be observed, apart from the previously mentioned trends of increased susceptibility of the CF strains against the combined treatments, it is very difficult to extract more common markers for the strains of each collection. The formation of three clusters was obtained, with two of them only being constituted by bacteremia-proceeding isolates. Nevertheless, the rest of bacteremia strains were clustered together with CF ones, showing that the correlations among the studied parameters seem weak. Moreover, a clear separation between early-late CF isolates was not obtained. All these circumstances could at least be partially explained by the complex heterogeneity of populations that have been shown to co-exist in the CF patient’s lungs, even constituting a single strain with the typical biofilm lifestyle cells but also with planktonic variants^48,49^.

## Discussion

In this work, the susceptibility to the innate humoral weapons targeting the PGN lysozyme and PGLYRPs (alone or in combination with the permeabilizing agent colistin^14,15^), has been characterized for the first time in *P. aeruginosa* clinical isolates from the two most relevant and complementary scenarios: acute (bloodstream) vs. chronic (CF lung) infections. The overall activity of these proteins *per se* was low in the majority of the strains, with some exceptions showing increased lysozyme susceptibility. In this regard, we propose the disruption of *mpl* gene (likely entailing a PGN recycling impairment) as explanation for the above-mentioned increased susceptibility in the strain PAFQ11-10. *mpl* encodes the UDP-N-acetylmuramate:L-alanyl-gamma-D-glutamyl-meso-diaminopimelate ligase that allows the recycling of tripeptides to contribute to the PGN monomers’ biosynthesis^11,50^. Mpl inactivation has been related with mild AmpC hyperexpression and β–lactam resistance, an obvious reason for its selection in CF^11,21,22,51^ and has been proposed as antibacterial target through the use of toxic alternate substrates^52^, data adding relevance to our findings.

We have recently observed that strains with impaired PGN recycling (KO mutants in AmpD-AmpDh2-AmpDh3, NagZ, AmpG) show increased susceptibility to lysozyme and PGLYRPs but only when subinhibitory colistin is added^14^. Thus, we hypothesize that at least some of the strains showing high susceptibility to the combined treatments could have mutations altering the PGN recycling. In this sense, although we have previously found the presence of polymorphisms in AmpDh2, AmpDh3, NagZ or AmpG in some of the strains used in this work (PAFQ6, PAFQ10, PAFQ15 and PAFQ24 pairs)^53–55^, the existence of inactivating mutations in these genes in clinical strains has barely been investigated elsewhere. Therefore, since the effect of these polymorphisms has not yet been determined, then it cannot be affirmed that the cause of their enhanced susceptibility is linked to a PGN recycling blockade. Conversely, the disruption of *mpl* in PAFQ11-10 likely impairs PGN recycling, at least regarding the tripeptide’s re-utilization.

What is also interesting about the PAFQ11-10 isolate is its high lysozyme susceptibility even without the need for permeabilization, which adds importance to its study as a source of therapeutic targets, namely *mpl* and/or additional ones. In fact, the existence of elements involved in PGN metabolism with implications as potential targets, such as the inactivation of *murA* and *murD* driving to a major attenuation of *P. aeruginosa* virulence, has been reported^56^. Nevertheless, it is still unclear whether the increased susceptibility linked to *mpl* loss is due to PGN structural defects significant enough to sensitize the bacterium in spite of the permeability barrier, and/or to a disorganized outer membrane facilitating the entrance of lysozyme (already described for other targets^28^), and/or to additional reasons. Similarly, it still needs to be determined whether the concomitant hyper-susceptibility of PAFQ11-10 to PGLYRPs + colistin is related to this *mpl* inactivation. However, the linkage *mpl*-PGLYRP2 susceptibility seems more plausible, given the PGN lytic activity that this protein displays, on the contrary of PGLYRP1 (allegedly not reaching the PGN in gram-negatives to exert its bactericidal activity)^57–60^.

Moreover, to the contrary of what happens for other variables during the adaptation to the CF niche such as mutation frequency^61^, antibiotic resistance^16^, etc.^27,62^, our results suggest that there is no general rule for lysozyme susceptibility evolution when comparing acute, and early/late chronic strains. Therefore, the lysozyme susceptibility patterns we display could be more related with the particularities of each patient of procedence. Thus, for instance, the host’s level of lysozyme activity (influenced by mucus osmolarity^63,64^, level of free docosahexaenoic acid^65^, degree of interaction with anionic biopolymers in the inflamed lung^66^ etc.) could have played a role in the selection of the mentioned phenotypes, obtained as a collateral effect of adaptation to the specific niche. A similar explanation could be given for the observed trends of increased susceptibility of late CF isolates against lysozyme/PLGYRPs + colistin. Moreover, our data suggest that adaptation to CF niche could cause collateral damages in the cell-wall entailing increases in susceptibility against lysozyme earlier (visible in initial isolates) than against PGLYRPs (only visible in late CF isolates).

The modest activity of lysozyme *per se* is not surprising, since it apparently needs a permeabilizer to exert its lytic activity on the gram-negative PGN, achieved through other actors *in vivo* (lactoferrin and/or defensins)^14,67–70^. In fact, the antipseudomonal activity of lysozyme in these works was shown to be modest, although the number of tested strains was very limited. Hence, our work shows that lysozyme resistance is not a constant, and that some attention-calling phenotypes of enhanced susceptibility can be found if analyzing large collections of clinical isolates.

Dealing with bacterial adaptation, in this case with regards to colistin resistance, in *P. aeruginosa*, it is well known that certain mutations cause the addition of phosphoethanolamine and/or 4-amino-4-deoxy-L-arabinose to lipid A within the LPS, which reduces the interaction with colistin^24,25,71^. We have used the *phoQ* mutant, displaying a notably increase on the colistin MIC^24–26^, to ascertain whether resistance mechanisms could impair the activity empowerment of the combined treatments. Our results show that the increase in the antipseudomonal power of lysozyme/PGLYRPs after colistin addition is not affected, enforcing the potential of these combinations as future therapeutic options. In fact, the adjuvant power of colistin over unconventional antibiotic combinations^72–74^ has been reported. Furthermore, although some works have suggested that colistin resistance could entail cross-resistance to antimicrobial peptides in *Enterobacter cloacae* and *Acinetobacter baumannii* based on LPS modifications^75–78^, this has never been proven for *P. aeruginosa*. As such, the existence of *P. aeruginosa* colistin-resistant mutants due to LPS total loss has not been proven either and conversely, mutations in genes involved in its synthesis, such as *galU*, *lptC* or *wapR*, significantly decrease the MICs^26^. These facts suggest that *P. aeruginosa* shows some particularities in the colistin resistance development of which we should take advantage, and mainly in the context we describe here by potentiating the effects of our cell-wall-targeting weapons.

With regards to serum, our results are in accordance with previous studies showing a fair predominance of serum-susceptible strains in CF patients^30,32,79–82^. The loss of O-antigen typically found on CF isolates (rendering non-typeable strains often reported to be serum-susceptible) is also in accordance with our results: 19 of our 24 CF isolates were non-typeable. But, precisely because of this we perhaps expected an overall higher degree of susceptibility in our CF isolates [less than the half could be considered susceptible (survival below 1%)], and even a trend to increased susceptibility when comparing early with late CF isolates. Nevertheless, some works have demonstrated that the existence of non-typeable strains (lacking O-antigen) proceeding from CF patients and showing serum-resistance was not that exceptional^83,84^. These studies also proved that the typeable isolates are not necessarily serum-resistant, features that some of our strains display. Moreover, the observation of a trend using two isolates (early-late) does not always ensure covering the necessary time lapse to visualize the selection of a certain phenotype. This is what may have happened with some of our CF isolates. This hypothesis has been previously suggested in other works dealing with additional adaptations during the CF process^27^. Thus, our results suggest questioning the classic conception of CF-proceeding strains being almost uniformly susceptible to serum^32,79–82^. In any case, as could be argued for the rest of treatments and phenotypes, the particularities of CF lung (poor diffusion of complement compounds and/or reduced level of immune activation enabled by the biofilm lifestyle and/or by the loss of O-chains, etc.) have been proposed to allow the selection of complement-susceptible phenotypes in exchange for other benefits achieved through adaptive mutations^81,85–87^. Otherwise, the need for LPS-linked resistance to killing and opsonization by complement to survive within the bloodstream^88–90^ has been highlighted, which obviously supports the results of high resistance of our bacteremia strains.

On the contrary, our results (the overall inflammatory capacity of CF strains was higher than that of bacteremia) would not be in line with works defending the selection of less inflammatory variants of LPS/PGN in CF strains in order to decrease immune activation and recognition, and thus ameliorate chronic persistence^91,92^. Our data would not agree either with those works claiming that the reduced inflammation found in CF strains is due to factors such as TTSS downregulation, alginate production or quorum-sensing-related genes mutations^93–95^. Conversely, several studies^45–47^ claim that the loss of inflammatory capacity is not a hallmark of chronic strains in contrast with virulence attenuation, which would support our results in this regard.

Although little information dealing with defensins’ antipseudomonal power has been published, it seems to be in line with our findings, given the potent activity we have shown *in vitro*^33–35,68,96^. These innate weapons seem to play an essential defensive role: its absence has been related to increased severity of infection in animal models^33–35^, whereas *P. aeruginosa* has been shown to induce the expression of defensins on epithelia^97^. Defensins have been classically thought to be bactericidal only through membrane permeabilization^37^, but recent works have suggested that not all the variants share a common mechanism of action^38^. In fact, the defensin we have taken as model, (β–defensin 1), has been shown to be incapable of permeabilizing the *E. coli* membrane although its antibacterial power is similar to that of other defensins proved to be membrane-permeabilizers^38,98^. Finally, the role of the LPS as a defense against these innate peptides has been previously shown for *Salmonella*^68^ but not for *P. aeruginosa*. In fact, our results seem to be in line with this, since some CF strains (e.g. the PAFQ24 pair), shown to be non-typeable and hence with probable loss of O-chains, were highly resistant against defensin treatment.

Altogether our results suggest that the generation of diverse phenotypes we display here is a very complex and multifactorial issue, in which several actors could be taking part. Therefore, each strain’s phenotype should be individually approached to ascertain the molecular basis, with it being very difficult to draw general trends. Thus, not only the bacterial adaptation to each patient and specific niche (bloodstream/CF lung) but also the complex modes of action of lysozyme and PGLYRPs could contribute to generate particularities among the studied treatments/strains. For instance, in the case of lysozyme, besides a classic PGN-degrading activity it has been bestowed with membrane-disturbing power^99–101^. On the other hand, PGLYRP1 (and PGLYRP3 and 4), is considered an inducer of multiple stresses driving to bacterial suicide independent of PGN lysis^57–60^. This complexity is illustrated by the lack of uniformity among strains regarding the susceptibility profiles against different treatments, a circumstance that must be emphasized and is easily visible in two of the most attention-calling strains in this work: PABA-12 and PAFQ11-10. Both strains were highly susceptible to PGLYRPs after permeabilization, but remained resistant to their action when colistin was absent. On the contrary, they were highly susceptible to lysozyme alone, suggesting that lysozyme penetrates better than PGLYRPs. Therefore, their enhanced susceptibility could be more related to defects on PGN structure than on permeability barrier. Nevertheless, a certain influence of this latter cannot be discarded either because, whereas PABA-12 only showed considerable resistance to PGLYPRs *per se* and osmotic shock, PAFQ11-10 was highly resistant to serum and defensin treatments. Thus, one alternative possibility could be that PABA-12 showed significant enough defects in the permeability barrier to sensitize the cells against almost all the treatments. This would be supported by the lack of agglutination regarding the serotyping of this strain, suggesting a truncated LPS which would obviously impair the permeability barrier. Nevertheless, the lack of agglutination cannot be the only cause, since none of the rest of non-typeable strains were as *pan-susceptible* as PABA-12 (Supplementary DataSet S1). On the contrary, the mechanistic basis for the susceptibility of PAFQ11-10 against lysozyme and PGLYRPs + colistin would be probably less related with permeability issues, and more related to PGN recycling impairments caused by the mentioned *mpl* mutation, that may not affect the serum/defensin susceptibility.

As additional evidence of this complexity, some other actors could be considered to influence the phenotypes against the treatments we characterize in this study. For instance, a potential impact of the antibiotic resistance profile (more specifically regarding β–lactams, given its obvious relatedness with the PGN biology), and/or the production of alginate (mucoid phenotype) over the susceptibility to the cell-wall-targeting proteins could exist. Nevertheless, when analyzing these two parameters, (Supplementary DataSet S1), no correlation between the mucoid phenotype (8 of our 24 CF isolates showed this phenotype) or the ceftazidime resistance (marker for β–lactam resistance) could be established with an increase/decrease of susceptibility to the studied treatments. Nevertheless, although in our assays the alginate hyperproduction seemed to confer no clear advantadges for bacterial survival, we cannot assume that the combinations we propose are not prone to sequestration by biofilm matrix in a chronically infected patient. Therefore, it iwould be an important issue to be also addressed in the future using appropriate models.

Moreover, it has been described that the CF-proceeding strains often display a lower elastase activity in comparison with those proceeding from acute infections^102,103^. To rule out the possibility of our CF strains having an increased susceptibility to lysozyme/PGLYRPs (+colistin) due to a diminished elastase production, we determined their elastase activity as previously described^104^. However, no significant differences among collections were found [median values of elastase activity with regards to PAO1 (considered 1); bacteremia: 0.83, early CF: 1.2, late CF: 0.82 (data not shown)] thus minimizing the potential impact of this feature over our final results.

Either way, a fact that could add potential and relevance to our findings is that PGLYRPs are elements to which the development of additional resistance by pathogens is considered unlikely^105^. This is because typical antibiotics have one primary target and mode of action, whereas PGLYRPs (and probably lysozyme) bind to different elements of bacterial envelopes and show different mechanisms of action (PGN degradation, permeabilization of membranes, etc.), which would make the selection of specific resistance mechanisms^105,106^ difficult. In fact, studies with natural and engineered antimicrobial peptides have been becoming a trend in recent years in the field of anti-biofilm activity and/or as alternative options *per se* or in combination with other antimicrobials in order to curb the threat of bacterial resistance increase to the classic antibiotics^107–111^. Thus, our work fits this expanding field, envisaged as an encouraging therapeutic strategy to defeat *P. aeruginosa* infections.

## Concluding Remarks

The encouraging results we show here enforce the idea that attacking some of the *P. aeruginosa* cell-wall biology-related elements (using subinhibitory colistin as permeabilizer or through targets weakening the cell-wall) to increase the activity of our innate humoral weapons targeting the PGN or being the adjuvant for the use of exogenous lysozyme/PGLYRPs to be administered as a combined treatment, could be a very promising therapeutic strategy. Moreover, there is scarce evidence of additional resistance development capacity *P. aeruginosa*. And, even more if we consider that the increase of activity of lysozyme/PGLYRPs seems to be independent of colistin resistance mechanisms. Our findings also indicate that *P. aeruginosa*, when adapting to CF niche, tends to accumulate changes that render the cell more susceptible to the cited innate proteins. Besides, our results suggest that many factors are involved in the generation of each phenotype, and hence, even when analyzing susceptibility against closely related immune weapons, each strain is a particular case and few general trends can be extracted. However, these facts are indeed convenient since almost each strain probably displays a very particular array of mutations in diverse targets driving to the final phenotype. This is well worth future study to reveal potential therapeutic targets, a very urgent need in the current panorama of antipseudomonal drugs shortage.

## Methods

### Bacterial strains and antimicrobial susceptibility testing

Besides the reference strains for comparative purposes (PAO1 and PA14), all the strains used in this work belong to two previously described collections of clinical isolates and are displayed in Data Set S1, together with their relevant features. First, twelve strains isolated from bacteremia patients and belonging to a collection from a Spanish multicenter study^112^ were included. These strains were selected in order to have representatives of hospitals from different areas of Spain, different profiles of antibiotic resistance^113^, and of the most relevant/prevalent clones (sequence types, STs). Second, we used twelve pairs of sequential isogenic isolates [early-late isolate, obtained with a difference of at least 3 years from a total of 10 patients (thus, from two of the patients, two pairs of isolates were obtained on each, at different time points)], proceeding from a previously published work^114^. When indicated, *P. aeruginosa* knockout mutants from the University of Washington Transposon Library were used, always in comparison with their originative wildtype strain (PAO1-UW)^23^. The susceptibility (minimal inhibitory concentration, MIC) against colistin (Sigma-Aldrich) was determined through broth microdilution as recommended by EUCAST (www.eucast.org).

### O-antigen Serotyping

Bio-Rad monoclonal antisera for the 16 more usually detected serotypes of *P. aeruginosa* were used to type the different isolates used in this study, following the manufacturer’s recommendations.

### Lysozyme susceptibility assays

The bactericidal activity of chicken egg white lysozyme (50000 units/mg protein; > 99% protein) (Sigma-Aldrich) was assessed in all the strains following previously described protocols^14^. A total of 1 × 10^6^ CFUs of each strain, proceeding from overnight LB broth cultures, were incubated in sodium phosphate buffer (10 mM [pH 7.0]) with 25 mg/L of lysozyme (in a total volume of reaction of 0.3 mL) for 1 h at 37 °C-180 rpm agitation, and quantified by serial plating at the beginning and end of incubation. The experiments were also performed with the addition of colistin as a permeabilizing agent, at a final sub-inhibitory concentration of 0.025 mg/L, in independent experiments. Additionally, the effect of colistin alone on the different strains was also studied, using the same procedure and buffer without adding lysozyme. All experiments were performed at least in triplicate.

### PGLYRPs susceptibility assays

The bactericidal activity of purified human PGLYRP1 or PGLYRP2 (AmsBio), was assessed in all the strains following previously described procedures^14^. Suspensions of approximately 1 × 10^5^ stationary phase CFUs were incubated 2 h at 180 rpm-37 °C in the assay buffer: 50 mg/L of the corresponding PGRP, 5 mM Tris-HCl buffer (pH 7.6), containing NaCl 150 mM, ZnSO4 5 µM, Glycerol 5% and LB broth 1%. The viable bacteria were quantified by serial plating on LB agar plates at the beginning and end of incubation. The experiments were also performed with the addition of colistin, at a final sub-inhibitory concentration of 0.025 mg/L, in independent experiments. Additionally, the effect of colistin alone on the different strains was also studied, using the same procedure and buffers without the addition of any PGLYRP. All experiments were performed at least in triplicate.

### Serum bacterial killing

Serum bactericidal assays were performed following previously described protocols^115^. Bacteria from overnight LB agar plates were resuspended in PBS with Ca and Mg (Sigma-Aldrich) to a final concentration of approx. 2.5 × 10^7^ CFU/mL; 80 µl of this suspension were mixed with 40 µl of PBS with Ca and Mg and 40 µl of nonimmune human serum (NHS) or heat-inactivated (HI) NHS. The samples were incubated at 37 °C for 30 min and then serially diluted and plated to determine the bacterial killing with regards to the number of bacteria in tubes with HI-NHS. Experiments were always performed at least in triplicate.

### Human β-Defensin 1 susceptibility assays

Susceptibility assays were performed following previously described protocols^116,117^ with slight modifications. Suspensions of approximately 1 × 10^5^ stationary phase CFUs of each strain were incubated 2 h at 180 rpm-37 °C in the assay buffer: 10 mM sodium phosphate buffer pH 7.4, with a final concentration of 10 µg/mL of human β-Defensin 1 (AmsBio), in a volume of reaction of 50 µL. Serial dilutions and plating at time point 0 and in the end of incubation were used to calculate the percentage of bacterial survival. These assays were performed at least in triplicate.

### Osmotic shock assays

The resistance of all the strains against hypo-osmotic shock was assessed following published protocols with slight modifications^29,118^. Overnight LB broth cultures were centrifuged and washed three times with double distilled water and afterwards resuspended in a final volume of 20 mL of double distilled water at a final concentration of 1 × 10^6^ CFUs/mL. The suspensions were incubated at room temperature for 24 hours with gentle agitation. The bacterial viability was quantified through colony count after serial dilutions and plating at the beginning and the end of incubation. The assay was performed at least three independent times per strain.

### Cell culture and cytotoxicity assays

The A549 human type II alveolar epithelial cell line was purchased from Cell Line Service and used between the passages 3 and 30. The cells were maintained in Dulbecco’s modified Eagle’s medium (DMEM) (Sigma-Aldrich) supplemented with 10% of heat-inactivated fetal bovine serum, 10 mM HEPES, 2 mM L-glutamine and 1X antibiotic-antimycotic solution (Biowest). Cells were seeded at approx. 1 × 10^5^ cells per well in 24-well plates the day before experiments. The day after, the cells were at approx. 80% of confluence, and were infected at a MOI of 100, following previously described protocols^45^. Briefly, bacteria were grown to log phase and diluted in RPMI 1640 medium (without phenol red and without fetal serum to avoid interferences with posterior assays) (Biowest) before infection. Afterwards, the old medium was removed, the wells washed with PBS, and the new medium containing the bacteria finally added to each well. The plates were then centrifuged (1000 g for 5 min) to synchronize the arrival of bacteria to the cells. After 3 hours incubation, the medium was collected and used to determine the cytotoxicity of each strain. For this purpose, the Lactate DesHydrogenase (LDH) release from the death cells was measured using the Cytotoxicity Detection Kit PLUS (Roche), following manufacturer’s instructions. These assays were performed with samples proceeding from at least 9 wells of cell culture plates (three wells from each of 3 independent plates) per strain.

### Inflammatory response

To assess the inflammatory response elicited by the different strains over the A549 cell cultures, the secretion of IL-8 interleukin was used as an indicator^45^. To infect the cells, the same protocol used for LDH release was followed, but a MOI of 5 was used instead of 100, to match the cytotoxicity levels of all the strains (always below 5%). The supernatants of cells after infection were used as samples and non-infected wells were used as basal controls. The Human IL-8/NAP-1 Instant ELISA kit (eBioscience-Affymetrix) was used following the manufacturer’s instructions. The IL-8 release assays were performed with samples proceeding from at least 9 wells of cell culture plates (three wells from each of 3 independent plates) per strain.

### Data analysis

The GraphPad Prism 5 software was used for graphical representation and statistical analysis. The variables were compared using the one-way ANOVA (with or without the *post hoc* Tukey’s multiple comparison test) or the Student *t* test as appropriate, being a *P* value of < 0.05 considered statistically significant.

For the hierarchical clustering of the strains, only the parameters shown to be significantly different among strains were considered. The unsupervised clustering of the data was performed (Z-score normalization, UPGMA, Euclidean distance), using the software HCE 3.5, available at http://www.cs.umd.edu/hcil/hce/ (University of Maryland).

## Supplementary information

DataSet 1

Supplementary Figures
