# Publisher Correction: Profiling the susceptibility of *Pseudomonas aeruginosa* strains from acute and chronic infections to cell-wall-targeting immune proteins

**DOI:** 10.1038/s41598-020-60494-5

**Published:** 2020-03-04

**Authors:** Gabriel Torrens, Isabel M. Barceló, Marcelo Pérez-Gallego, Maria Escobar-Salom, Sara Tur-Gracia, Marta Munar-Bestard, María del Mar González-Nicolau, Yoandy José Cabrera-Venegas, Estefany Nayarith Rigo-Rumbos, Gabriel Cabot, Carla López-Causapé, Estrella Rojo-Molinero, Antonio Oliver, Carlos Juan

**Affiliations:** Servicio de Microbiología and Unidad de Investigación, Hospital Universitari Son Espases-Institut de Investigació Sanitària de Balears (IdISBa), Palma, Spain

Correction to: *Scientific Reports* 10.1038/s41598-019-40440-w, published online 05 March 2019

A Supplementary File containing Figures S1, S2, S3, S4 and S5 was omitted from the original version of this Article. This has been corrected in the HTML version of the Article; the PDF version was correct at time of publication.

