# Supplementary material for: Profiling the susceptibility of Pseudomonas aeruginosa strains from acute and chronic infections to cell-wall-targeting immune proteins
Source: Sci Rep. 2019 Mar 5;9:3575. doi: 10.1038/s41598-019-40440-w (PMC6401076; doi:10.1038/s41598-019-40440-w)
Supplement: Supplementary file 2 — Supplementary Figures [file 41598_2019_40440_MOESM2_ESM.pdf]

Profiling the susceptibility of *Pseudomonas aeruginosa* strains from acute and chronic infections to cell-wall-targeting immune proteins.

Gabriel Torrens[1], Isabel M. Barceló[1], Marcelo Pérez-Gallego[1], Maria Escobar-Salom[1], Sara Tur-Gracia[1], Marta Munar-Bestard[1], María del Mar González-Nicolau[1], Yoandy José Cabrera-Venegas[1], Estefany Nayarith Rigo-Rumbos[1], Gabriel Cabot[1], Carla López-Causapé[1], Estrella Rojo-Molinero[1], Antonio Oliver[1] & Carlos Juan[1].

[1]Servicio de Microbiología and Unidad de Investigación, Hospital Universitari Son Espases-Institut de Investigació Sanitària de Balears (IdISBa), Palma, Spain.

SUPPLEMENTARY INFORMATION

Data Set S1. Main features of the strains studied in this work. The strains are organized in two sheets: bacteremia (including also the reference strains and the used knockout mutants from the UW Transposon Library) vs CF strains (displayed in pairs of early-late isolates). The Data Set shows all the relevant features of the strains, including molecular epidemiology, serotyping, presence of mucoid phenotype, antibiotic resistance patterns (specifying the ceftazidime resistance phenotype, as a marker for β–lactam resistance), colistin MICs and finally, the mean value ± SD from each strain (obtained from at least three independent experiments of each parameter) regarding the bacterial survival after the following treatments: lysozyme, lysozyme plus colistin, PGLYRP1, PGLYRP2 (alone or plus colistin), colistin (in lysozyme buffer), colistin in PGLYRP's assay buffer, osmotic shock, β-defensin 1, and serum. The fold-reduction in bacterial survival after addition of colistin, with regards to treatment with lysozyme or PGLYRPs treatments alone is also shown (blue columns). The values of pro-inflammatory (IL-8 release, MOI 5) and cytotoxic (LDH release, MOI 100) effects on A549 cell culture are also displayed.

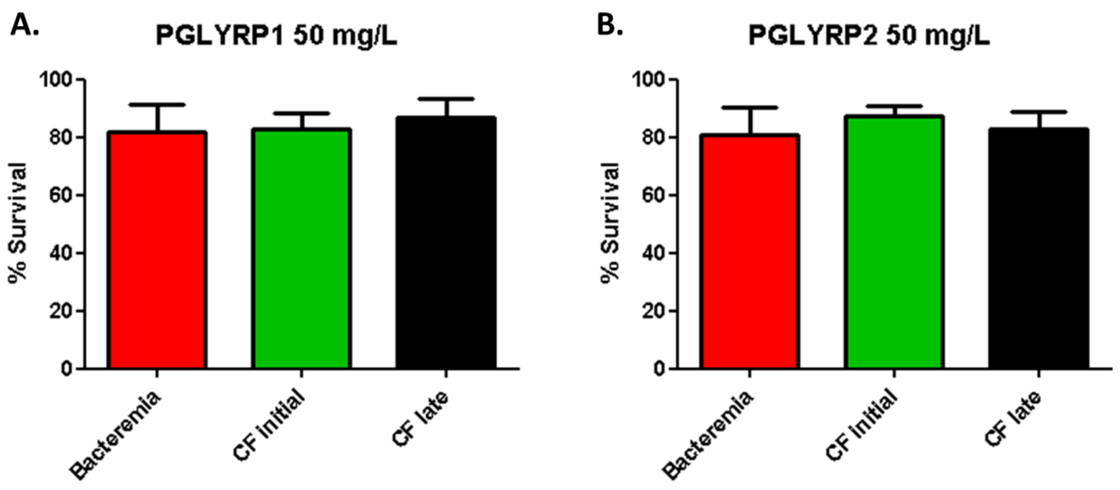

Figure S1. Survival of the *P. aeruginosa* strains from the collections studied after treatment with PGLYRPs. Incubations with PGLYRP1 (A) or PGLYRP2 (B) were performed as explained in materials and methods, and the survival percentage was calculated with regards to the initial inoculum. The columns represent the mean of bacterial survival of each collection together with the SD, represented by the error bars. The differences among bacteria treated with PGLYRPs and controls (bacteria incubated in the assay buffer without protein) were statistically significant in all the cases, with a slight growth in the control tubes (bacterial survival > 100%, $P < 0.05$, data not shown).

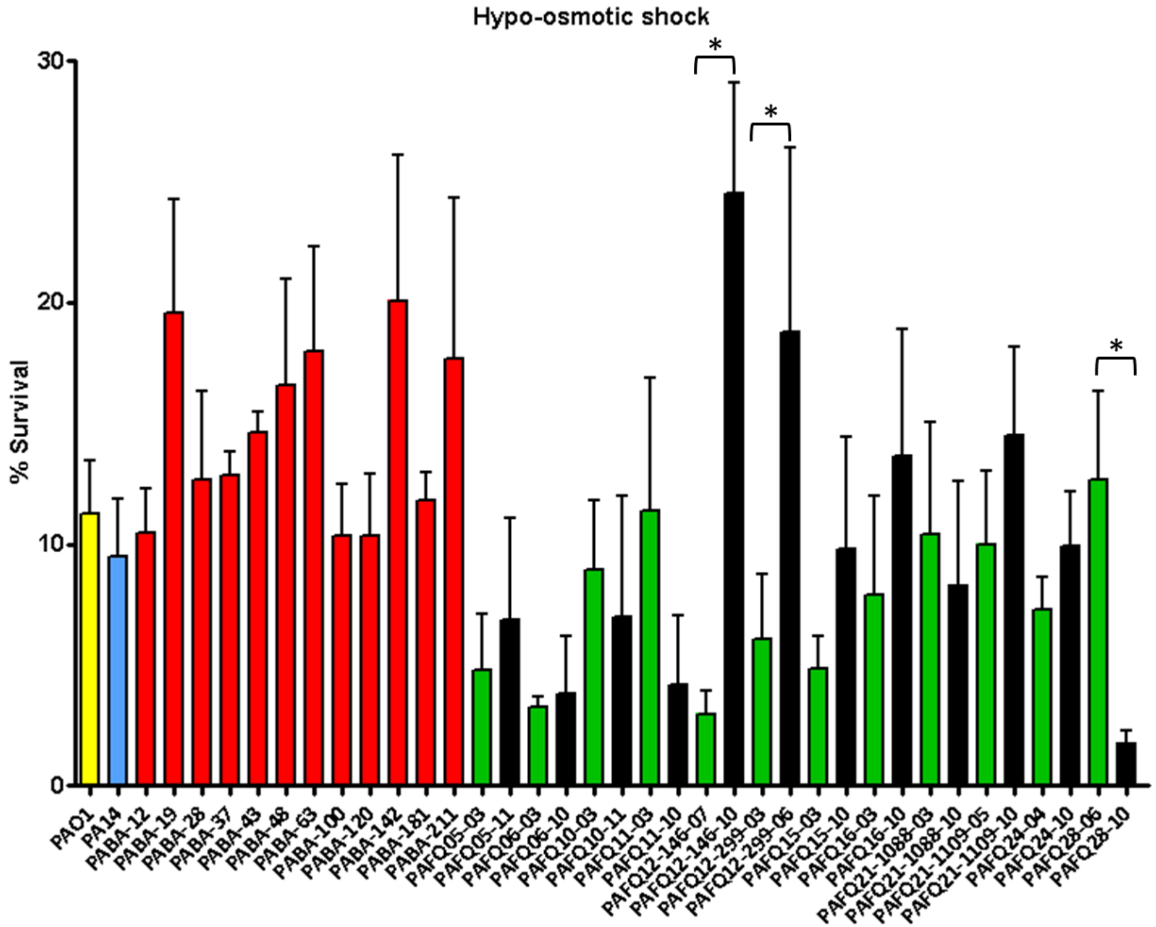

P value	Collections			Parameters	
Collections	Bacteremia	Early CF	Late CF	Median	Range
Bacteremia	NA	<0.0001*	0.06	13.7	10.3–21.1
Early CF	<0.0001*	NA	0.29	7.6	2.9 -12.6
Late CF	0.06	0.29	NA	9.0	1.75–24.5

Figure S2. Survival rates of the *P. aeruginosa* clinical strains studied after hypo-osmotic shock assay. Incubation was performed as explained in materials and methods ($1x10^6$ CFUs/mL of each strain in a total volume of 20 mL of double distilled water, 24h, room temperature with gentle agitation) and the survival percentage was calculated with regards to the initial inoculum. Each column represents the mean value of at least three independent assays for each specific strain, whereas the error bar represents the standard deviation (SD). In the CF pairs of isogenic isolates, the green columns correspond to early and the black to late isolates, respectively. All the bacteremia strains are displayed with red columns. The asterisks over the bars indicate an statistically significant difference between the early/late isolates in the specific pair(s) of CF strains, $P < 0.05$ in the One-way ANOVA with post hoc Tukey's multiple comparison test.

The box below displays the statistical parameters of the three collections of strains (bacteremia, early CF isolates and late CF isolates) regarding the survival after osmotic shock assay. The performed *t* tests were two-tailed in all the cases; only when comparing the early with late isolates within CF collection, a paired test was applied. *A P value < 0.05 was considered statistically significant. NA: Not applicable.

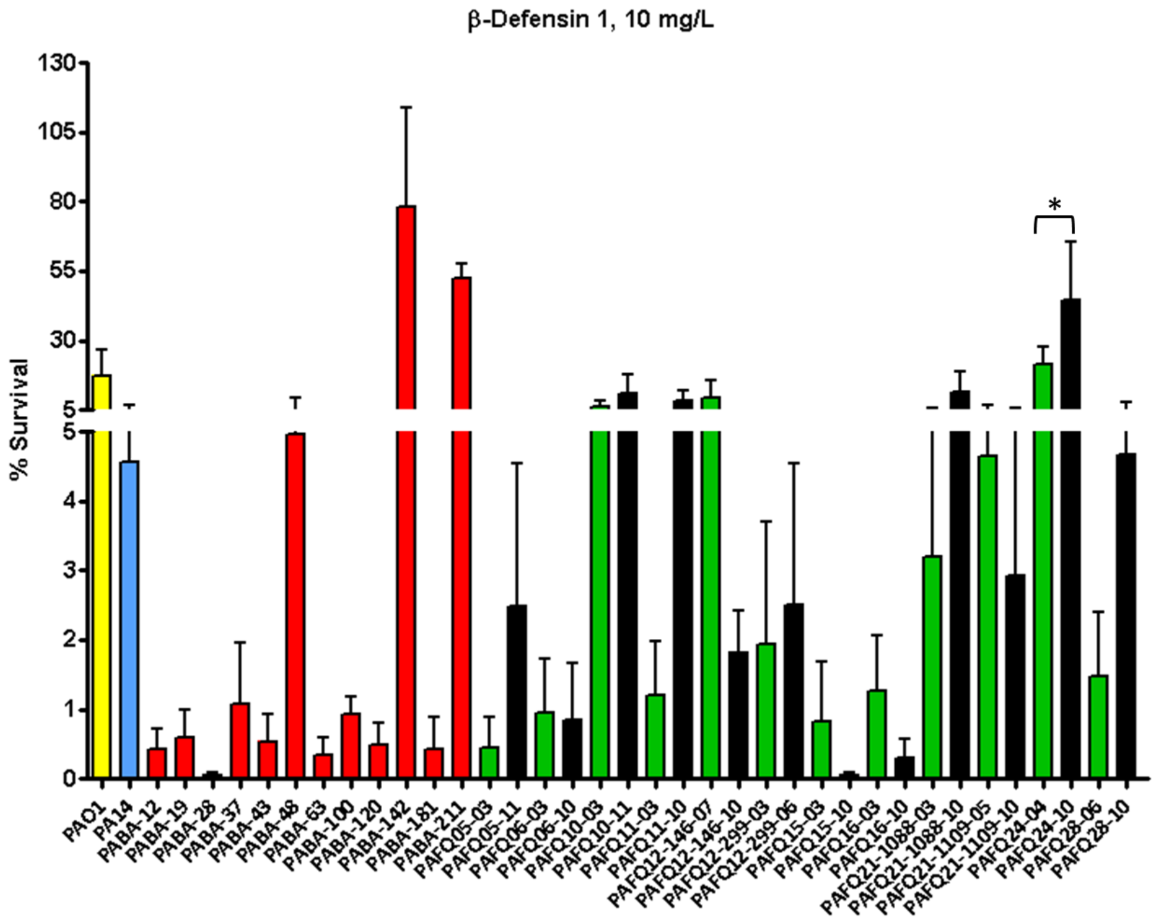

P value	Collections			Parameters	
**Collections**	**Bacteremia**	**Early CF**	**Late CF**	**Median**	**Range**
**Bacteremia**	NA	0.35	0.61	0.57	0.05–77.9
**Early CF**	0.35	NA	0.17	1.7	0.45-21.4
**Late CF**	0.61	0.17	NA	2.7	0.05–44.5

Figure S3. Survival rates of the *P. aeruginosa* clinical strains studied after treatment with human β–Defensin 1. Incubation was performed as explained in materials and methods ($1x10^5$ CFUs of each strain, 2h at 180rpm-37ºC), and the survival percentage was calculated with regards to the initial inoculum. Each column represents the mean value of at least three independent assays for each specific strain, whereas the error bar represents the standard deviation (SD). In the CF pairs of isogenic isolates, the green columns correspond to early and the black to late isolates, respectively. All the bacteremia strains are displayed with red columns. The asterisks over the bars indicate an statistically significant difference between the early/late isolates in the specific pair(s) of CF strains, $P < 0.05$ in the One-way ANOVA with post hoc Tukey's multiple comparison test. The differences among bacteria treated with human β–Defensin 1 and controls (bacteria incubated in the assay buffer without protein) were statistically significant in all the cases, with a slight growth in the control tubes (bacterial survival > 100%, $P < 0.05$, data not shown).

The box below displays the statistical parameters of the three collections of strains (bacteremia, early CF isolates and late CF isolates) regarding the survival after β–Defensin 1 treatment. The performed *t* tests were two-tailed in all the cases; only when comparing the early with late isolates within CF collection, a paired test was applied. *A P value < 0.05 was considered statistically significant. NA: Not applicable.

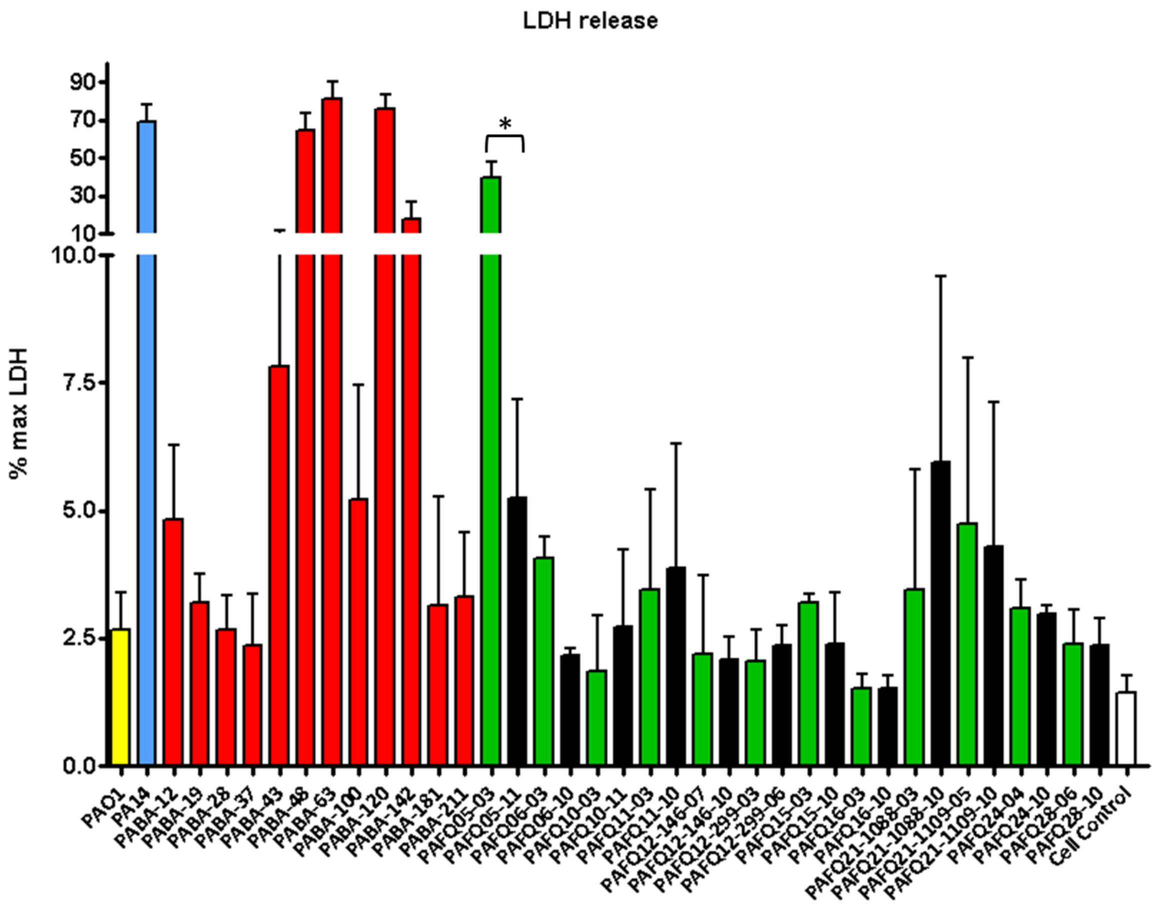

P value	Collections			Parameters	
Collections	Bacteremia	Early CF	Late CF	Median	Range
Bacteremia	NA	0.094	0.042*	5.22	2.35-80.9
Early CF	0.094	NA	0.35	3.15	1.5-39.4
Late CF	0.042*	0.35	NA	2.54	1.5-5.9

Figure S4. Cytotoxicity of the *P. aeruginosa* clinical strains studied. Determination of the LDH release by the infected cells was performed as explained in materials and methods (MOI 100, 3 hours). The released LDH is expressed as percentage of the maximum level released by a well of lysed confluent A549 cells. Each column represents the mean value of at least three independent assays for each specific strain, whereas the error bar represents the standard deviation (SD). In the CF pairs of isogenic isolates, the green columns correspond to early and the black to late isolates, respectively. All the bacteremia strains are displayed with red columns. The asterisks over the bars indicate an statistically significant difference between the early/late isolates in the specific pair(s) of CF strains, $P < 0.05$ in the One-way ANOVA with post hoc Tukey's multiple comparison test.

The box below displays the statistical parameters of the three collections of strains (bacteremia, early CF isolates and late CF isolates) regarding their cytotoxic capacity. The performed *t* tests were two-tailed in all the cases; only when comparing the early with late isolates within CF collection, a paired test was applied. *A P value < 0.05 was considered statistically significant. NA: Not applicable.

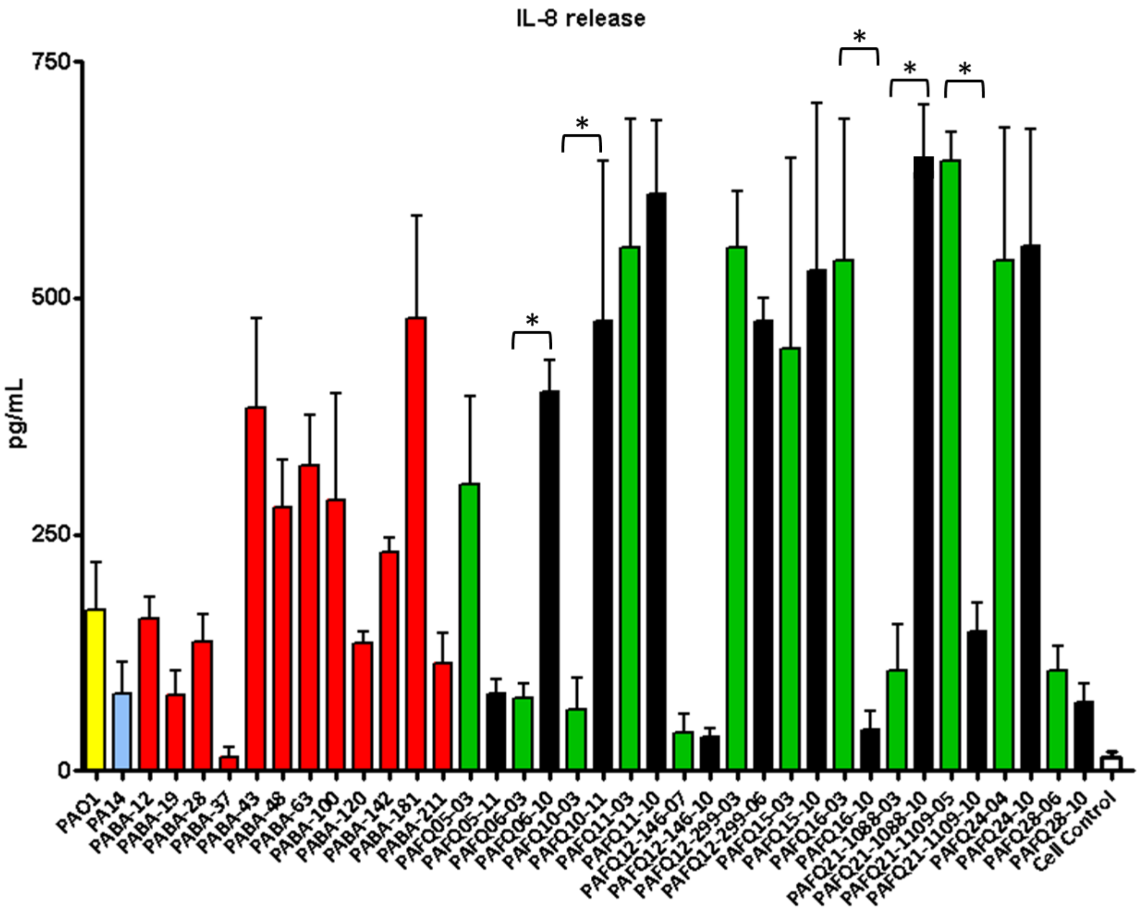

***P* value**	**Collections**			**Parameters**	
**Collections**	**Bacteremia**	**Early CF**	**Late CF**	**Median**	**Range**
**Bacteremia**	NA	0.18	0.24	195.4	17.8-468.7
**Early CF**	0.18	NA	0.93	349.5	28.9-647.3
**Late CF**	0.24	0.93	NA	421.6	36.3-656.3

Figure S5. Pro-inflammatory activity of the *P. aeruginosa* clinical strains studied. The determination of IL-8 release was performed as explained in materials and methods (MOI 5, 3 hours). The LDH release was proved to be below 5% (data not shown) for all the strains using this reduced MOI, in order to eliminate the confusion factor that the cell death could entail over the elicited inflammatory response. Each column represents the mean value of at least three independent assays for each specific strain, whereas the error bar represents the standard deviation (SD). In the CF pairs of isogenic isolates, the green columns correspond to early and the black to late isolates, respectively. All the bacteremia strains are displayed with the red columns. The asterisks over the bars indicate an statistically significant difference between the early/late isolates in the specific pair(s) of CF strains, $P < 0.05$ in the One-way ANOVA with post hoc Tukey's multiple comparison test.

The box below displays the statistical parameters of the three collections of strains (bacteremia, early CF isolates and late CF isolates) regarding the pro-inflammatory capacity. The performed *t* tests were two-tailed in all the cases; only when comparing the early with late isolates within CF collection, a paired test was applied. *A P value < 0.05 was considered statistically significant. NA: Not applicable.
